# Functional Evaluation of *Bacillus subtilis* DCP04 from Korean Fermented Soybean Paste: A Potential Probiotic Strain for Polyethylene Degradation and Adsorption

**DOI:** 10.3390/foods14193328

**Published:** 2025-09-25

**Authors:** Gyeong-Hwan Kim, Haemin Jeong, Injun Jung, Myounghyun Choi, Jong-Hoon Kim

**Affiliations:** 1 Department of Biotechnology, Pukyong National University, Busan 48513, Republic of Korea; cjstk6832@pukyong.ac.kr; 2 Decomposition Co., Ltd., Pohang 37583, Republic of Korea; jhmlina@decomposition.co.kr (H.J.); ij.jung@decomposition.co.kr (I.J.); light@decomposition.co.kr (M.C.)

**Keywords:** polyethylene, bio-adsorption, biodegradation, probiotics, *Bacillus subtilis*

## Abstract

Micro- and nanoplastics (MPs and NPs) are recognized as emerging contaminants posing potential risks to human health. Recent evidence highlights the potential of food-grade microbial strains to bind these particles and facilitate their removal, suggesting a promising probiotic-based strategy for mitigating their adverse health effects. This study investigated the adsorption and biodegradation capabilities of *Bacillus subtilis* DCP04, a strain isolated from Korean fermented soybean paste, cheonggukjang, on low-density polyethylene (LDPE) particles. Biofilm formation assays and morphological observations confirmed the strain’s ability to adhere to the surface of LDPE. Subsequent experiments demonstrated that DCP04 effectively adsorbed LDPE particles in a size-, time-, and concentration-dependent manner. This interaction induced significant morphological changes and increased hydrophilicity on the polymer surface. Furthermore, a positive correlation was observed between the activities of laccase and manganese peroxidase and a measurable weight loss in LDPE films, suggesting direct enzymatic involvement in polymer degradation. Crucially, the DCP04 strain also met key safety and functional criteria for use as a probiotic. These findings highlight the potential of DCP04 strain as a functional probiotic agent for mitigating the accumulation of MPs and NPs within the human body.

## 1. Introduction

Plastics are synthetic polymers, engineered with various additives to achieve desired functionalities, making them indispensable in modern society and industrial applications [1]. This widespread utilization across sectors such as industry, agriculture, and transportation has driven a continuous rise in global production. In 2021, production reached approximately 390.7 million tons—an increase of 15.2 million tons from 2020 and 25.5 million tons from 2018 [2]. Among the most prevalent type, polyethylene (PE), particularly its low-density (LDPE) and high-density (HDPE) forms, dominates the market and is predominantly used for single-use packaging materials like films and containers [3].

Humans are exposed to micro- and nanoplastics (MPs and NPs) through various pathways, including ingestion, inhalation, and dermal contact [4]. Upon entry into the body, these particles can induce a range of toxicological effects such as oxidative stress, chronic inflammation, endocrine disruption, and genotoxicity, potentially impairing reproductive and developmental functions. Such effects are linked to elevated risks of cancers, respiratory diseases, and adverse fetal outcomes [5]. Given that the primary route of exposure is the ingestion of contaminated food and water, the gastrointestinal (GI) tract is a major site of MP and NP accumulation and toxicity [6]. The accumulation of these particles can inflict direct physical damage on the intestinal lining, leading to inflammation, epithelial cell disruption, and a compromised mucosal barrier [7]. This damage increases intestinal permeability—a condition often termed “leaky gut”—allowing harmful substances and even the plastic particles themselves to translocate into systemic circulation [8]. Furthermore, MPs and NPs can disrupt the delicate balance of the gut microbiota, a state known as dysbiosis. This imbalance is characterized by a reduction in beneficial bacteria and a proliferation of opportunistic pathogens, which impairs metabolic functions. For instance, microbial alterations can decrease the production of essential metabolites like short-chain fatty acids (SCFAs), thereby compromising digestive processes and immune regulation [9,10]. The persistent presence of these foreign particles also triggers chronic immune activation and oxidative stress within the gut, elevating pro-inflammatory cytokine levels and causing sustained tissue damage [11]. This prolonged inflammation contributes not only to local GI disorders but also to systemic metabolic and immune dysfunctions [12]. In summary, the accumulation of ingested plastics compromises the gut’s physical and microbiological barriers, posing substantial risks to overall human health [13].

The significant health risks posed by the bioaccumulation and toxicity of MPs and NPs necessitate the development of innovative removal and management strategies [14]. Among the potential solutions, biological approaches using microorganisms and their enzymes have emerged as a particularly promising avenue due to their potential for sustainability and efficiency [15]. A novel application of this approach focuses on the use of probiotics to mitigate plastic exposure in vivo. Recent evidence suggests that specific probiotic strains, particularly those with Generally Recognized as Safe status like *Lacticaseibacillus paracasei* and *Lactiplantibacillus plantarum*, can adsorb microplastics within the GI tract. This binding action is hypothesized to reduce the bioavailability of the particles and facilitate their excretion, thereby offering a potential strategy to counteract microplastic-induced gut dysbiosis and protect host health [16].

Among potential probiotic candidates, *Bacillus subtilis* is particularly noteworthy. It is widely recognized for its capacity to modulate gut microbiota, enhance immune responses, and serve as an effective alternative to antibiotics [17]. Furthermore, various *B. subtilis* strains possess remarkable detoxification capabilities, such as reducing toxic compounds, and are well-known for forming robust biofilms that mediate interactions with diverse materials [18,19,20]. This unique combination of established probiotic functions and strong surface-adhering properties makes *B. subtilis* an ideal candidate for targeting MPs and NPs within the gut.

Cheonggukjang, a traditional Korean fermented soybean paste, was specifically selected as the isolation source for this study. This food is known to be particularly rich in *Bacillus* species, most notably *B. subtilis*. As previously documented, various *B. subtilis* strains are renowned for their ability to produce a wide array of robust enzymes and to form resilient biofilms. These characteristics, strong enzymatic activity and superior adhesion capabilities, are considered critical for the effective adsorption and biodegradation of recalcitrant polymers like PE. Therefore, cheonggukjang was identified as an optimal source for isolating novel bacterial candidates with high potential for plastic degradation.

Therefore, the primary aim of this study was to isolate and characterize a novel *Bacillus subtilis* strain, designated DCP04, from the traditional Korean fermented soybean paste, cheonggukjang, and to comprehensively evaluate its potential as a probiotic agent for mitigating plastic particle pollution. To achieve this, the following specific objectives were established: (1) to confirm the safety profile and key probiotic properties of the isolated strain; (2) to investigate its ability to adsorb LDPE particles across various sizes and conditions; (3) to assess its biodegradative capability on LDPE films by analyzing enzymatic activity, weight loss, and resultant chemical and morphological surface modifications.

## 2. Materials and Methods

### 2.1. Materials

LDPE powders with particle sizes of 25 μm, 200 nm, 100 nm, and 50 nm were procured from Decomposition Co., Ltd. (Pohang-si, Republic of Korea). LDPE films were purchased from Goodfellow (Cambridge, UK). Prior to use, all LDPE materials were sterilized. The powders and films were immersed in 70% (*v*/*v*) ethanol for 2 h, followed by air-drying in a laminar flow hood under aseptic conditions. The dried powder was subsequently subjected to UV irradiation for 2 h for further sterilization. Commercially available cheonggukjang was purchased from Inkun Inc. (Gwangju city, Republic of Korea).

### 2.2. Screening, Isolation, and Identification

The cheonggukjang sample was serially diluted in sterile phosphate-buffered saline (PBS). Aliquots of the dilutions were spread onto a basal salt agar medium (BSAM) consisting of (per liter): 12.8 g Na_2_HPO_4_·7H_2_O, 3 g KH_2_PO_4_, 0.5 g NaCl, 1 g NH_4_Cl, 2 mM MgSO_4_, and 0.1 mM CaCl_2_. The medium was supplemented with 0.1% (*w*/*v*) sterile LDPE powder as the sole carbon source [21]. Following incubation at 37 °C for 48 h, colony that formed a clear halo, indicating potential degradation activity, was selected for further analysis. For molecular identification, genomic DNA was extracted from the pure isolate using the AccuPrep^®^ Genomic DNA Extraction Kit (Bioneer, Daejeon, Republic of Korea) per the manufacturer’s protocol. The 16S rRNA gene was amplified via polymerase chain reaction using universal primers 27F (5′-AGAGTTTGATCCTGGCTCAG-3′) and 1492R (5′-AAGTCGTAACAAGGTAACC-3′). The resulting amplicon was sequenced, and the sequence was used for phylogenetic identification via the National Center for Biotechnology Information (NCBI) database. Phylogenetic analysis was conducted using CLUSTAL X (version 2.1) for sequence alignment and MEGA X software (version 11.0) to construct a phylogenetic tree with the neighbor-joining algorithm and the Jones–Taylor–Thornton model [22,23].

### 2.3. Characterization of Probiotic Properties

#### 2.3.1. Hemolytic Activity

The hemolytic activity of the isolate, designated DCP04, was assessed on blood agar plates. The medium was prepared by supplementing sterile Blood Agar Base with 5% (*v*/*v*) defibrinated sheep blood (Kisan Bio, Seoul, Republic of Korea). The DCP04 strain was streaked onto the plates and incubated at 30 °C for 24 h [24]. *Staphylococcus aureus* ATCC 19615 and *Pseudomonas aeruginosa* ATCC 27853 were used as positive controls for *β*-hemolysis, while *Lacticaseibacillus rhamnosus* GG ATCC 53103 served as the non-hemolytic negative control [25]. Following incubation, plates were visually inspected for zones of hemolysis.

#### 2.3.2. Biogenic Amine Production

The potential for biogenic amine production was assessed by evaluating amino acid decarboxylase activity. The DCP04 strain was streaked onto bromocresol purple agar (Sigma-Aldrich, St. Louis, MO, USA) containing either lysine or arginine and incubated at 37 °C for 24–48 h. A color change in the medium from yellow to purple would indicate a positive result [26,27].

#### 2.3.3. Aggregation Assays

For both auto- and co-aggregation assays, the DCP04 strain was cultured to the mid-logarithmic growth phase (OD_600_ ≈ 0.8). Pathogenic strains (*S. aureus* ATCC 25923, *Escherichia coli* ATCC 25922, *P. aeruginosa* ATCC 27853) were cultured to the same optical density under their optimal conditions [28]. Cells were harvested by centrifugation (6000× *g*, 10 min, room temperature), washed twice with sterile PBS, and resuspended in PBS to a final OD_600_ of 0.8. For auto-aggregation, 1 mL of the DCP04 suspension was incubated in a microcentrifuge tube at 37 °C without agitation for 1, 2, 4, and 24 h. For co-aggregation, equal volumes (0.5 mL) of the DCP04 suspension and a pathogen suspension were mixed and incubated under the same conditions. At each time point, 100 µL was carefully collected from the upper layer of the suspension to measure the OD_600_.

The aggregation percentages were calculated using Equations (1) and (2) [29], where for auto-aggregation, *A*_0_ is the initial absorbance and *A_t_* is the absorbance at time *t*. For co-aggregation, *A_B_* and *A_P_* represent the initial absorbances of the probiotic and pathogen alone, respectively, and *A_mix_* is the absorbance of their mixture at time *t*:

(1) $$\text{Auto-aggregation}\;(\%)\;=\;\left[1-\frac{A_{t}}{A_{0}}\right]\times 100$$

(2) $$\text{Co-aggregation}\;(\%)=\frac{\frac{A_{B}+A_{P}}{2}-Amix}{\frac{A_{B}+A_{P}}{2}}\times 100$$

### 2.4. Enzyme Activity Assessment

For all enzyme assays, *Bacillus subtilis* ATCC 6051 was used as a type strain control.

#### 2.4.1. Laccase Activity

The DCP04 strain was cultured to the logarithmic phase (OD_600_ ≈ 2.0), and cells were harvested by centrifugation (10,000× *g*, 10 min, 4 °C). The cell pellet was washed twice with PBS and resuspended to create a cell suspension. Laccase activity was determined spectrophotometrically using guaiacol as the substrate [30]. The reaction mixture contained 2.4 mL of 100 mM guaiacol (in 0.1 M sodium acetate buffer, pH 5.0) and 0.6 mL of the cell suspension. The increase in absorbance at 470 nm was monitored at 25 °C. One unit (U) of laccase activity was defined as the amount of enzyme required to oxidize 1 μmol of guaiacol per minute [31].

#### 2.4.2. Manganese Peroxidase (MnP) Activity

Cell-free supernatant was obtained by centrifuging the DCP04 culture (10,000× *g*, 10 min, 4 °C). MnP activity was determined by monitoring the formation of Mn(III)–malonate complexes at 270 nm [32,33]. The reaction mixture (4.0 mL total) contained 3.7 mL of 50 mM sodium malonate buffer (pH 4.5), 0.1 mL of 4 mM MnSO_4_, 0.1 mL of 4 mM H_2_O_2_, and 0.1 mL of the supernatant. The reaction was incubated at 30 °C for 5 min. One unit (U) of MnP activity was defined as the amount of enzyme that produces 1 μmol of Mn(III) per minute, calculated using the molar extinction coefficient (ε270 = 11,590 M^−1^ cm^−1^).

### 2.5. LDPE Particle Adsorption by DCP04

#### 2.5.1. Biofilm Formation Assay

The capacity of the DCP04 strain to form biofilms was quantified using a crystal violet staining assay, adapted from previously described methods [26,34]. A bacterial suspension, standardized to a turbidity equivalent of a 0.5 McFarland standard, was prepared. Then, 1 µL of this suspension was inoculated into the wells of a 96-well plate, each containing 200 µL of sterile Tryptic Soy Broth (TSB; BD Difco, Franklin Lakes, NJ, USA). Wells with uninoculated TSB served as the negative control. The plate was incubated at 30 °C for 24 h. After incubation, non-adherent cells were removed by washing the wells three times with PBS. The plate was then air-dried for 30 min. The attached biofilms were stained with 0.1% (*w*/*v*) crystal violet solution for 30 min at room temperature. Excess stains were removed by washing three times with PBS, followed by another 30-min drying period. The bound dye was solubilized in 95% (*v*/*v*) ethanol, and the absorbance was measured at 590 nm using a microplate reader. The experiment was performed in quintuplicate.

#### 2.5.2. LDPE Adsorption Efficiency

The adsorption of LDPE nanoparticles (NPs) by the DCP04 strain was evaluated based on a modified protocol [35]. Cells from a 48 h culture were harvested, washed, and resuspended in PBS to a concentration of 5 × 10^9^ CFU/mL. A stock suspension of LDPE NPs (1 mg/mL) was prepared in distilled water and diluted to a working concentration of 20 µg/mL. The bacterial suspension was then mixed with the NP working solution and incubated at 37 °C for 4 h. Following incubation, the mixture was centrifuged (6000× *g*, 15 min, 4 °C) to separate the bacterial cells from the supernatant containing unbound NPs. The concentration of unbound NPs in the supernatant was quantified using a NanoSight NS300 instrument (Malvern Panalytical, Malvern, UK). The adsorption rate was calculated using Equation (3):

(3) $$\mathrm{Adsorption}\;\mathrm{rate}\;(\%)\;=\;\left[\frac{C_{control}-C_{sample}}{C_{control}}\right]\times 100\%$$

where *C_control_* is the NP concentration in the control group (without bacteria) and *C_sample_* is the NP concentration in the supernatant of the experimental group.

To assess the effects of different parameters on adsorption efficiency, three sets of experiments were conducted:

▪ Time dependency: An NP suspension (200 nm, 20 µg/mL) was incubated with DCP04 for 3, 6, 9, and 12 h.
▪ Size dependency: NP suspensions (50, 100, and 200 nm; 20 µg/mL) were incubated with DCP04 for 4 h.
▪ Concentration dependency: NP suspensions (200 nm) at concentrations of 10, 20, 50, and 100 µg/mL were incubated with DCP04 for 4 h.

#### 2.5.3. Morphological Observation of Adsorption

The interaction between the DCP04 strain and LDPE particles was visualized using field emission scanning electron microscopy (FE-SEM) and confocal laser scanning microscopy (CLSM).

For FE-SEM, bacterial cells, following the adsorption assay described in Section 2.5.2, were washed with PBS, fixed with 2.5% glutaraldehyde, dehydrated using a graded ethanol series, and freeze-dried. The dried samples were mounted and sputter-coated with platinum before observation with an FE-SEM (MAGNA, TESCAN, Brno, Czech Republic).

For CLSM, LDPE particles were first stained with Nile red (1 µg/mL in methanol). The stained LDPE particle suspension (10 µg/mL) was then added to a bacterial pellet (1 mL, 5 × 10^9^ CFU/mL) and incubated at 37 °C for 6 h. The interaction was subsequently observed using a CLSM system (DM2500, Leica Microsystems, Wetzlar, Germany). A bacterial suspension without LDPE particles served as the control [35].

### 2.6. Biodegradation of LDPE Film by DCP04

#### 2.6.1. Weight Loss Measurement

The biodegradation of LDPE films (1 × 1 cm, 0.15 mm thickness) was quantified by measuring weight loss, following a modified procedure [36]. The films were pre-treated by washing with double-distilled water, soaking in 70% ethanol (30 min), treating with 0.7% (*v*/*v*) Tween 80 and 1% (*v*/*v*) bleach solution (30 °C, 1 h), and finally treating with 30% hydrogen peroxide (60 °C, 1 min). After sterilization, the films were dried overnight and their initial weights (*W_i_*) were recorded.

Sterilized films were incubated at 30 °C for 14 days under three conditions: (1) TSB medium only (negative control), (2) TSB inoculated with *B. subtilis* ATCC 6051, and (3) TSB inoculated with *B. subtilis* DCP04. After incubation, the films were removed and washed sequentially with 2% sodium dodecyl sulfate, 70% ethanol, and distilled water to remove any attached biomass. The films were then dried at 60 °C for 1 h, and their final weights (*W_f_*) were recorded. The percentage of weight loss was calculated using Equation (4):

(4) $$\mathrm{Weight}\;\mathrm{loss}\;(\%)\;=\;\frac{\left(W_{i}-W_{t}\right)}{W_{i}}\times 100\%$$

#### 2.6.2. Surface Morphology Analysis

Surface changes on the LDPE films post-incubation were examined using FE-SEM. After the 14-day biodegradation assay, film samples were prepared for SEM as described in Section 2.5.3. An LDPE film maintained under control conditions (no bacteria) was used as a comparative control [37].

#### 2.6.3. FT-IR

Changes in the chemical structure of the LDPE films were analyzed using Fourier-transform infrared (FT-IR) spectroscopy. After the 14-day assay, the films were freeze-dried. FT-IR spectra were recorded from 4000 to 650 cm^−1^ with a resolution of 4 cm^−1^. An untreated LDPE film served as the control [37].

### 2.7. Statistical Analysis

All experiments were performed in triplicate. Statistical analyses were conducted using SPSS 21.0 software. The data were analyzed by one-way analysis of variance (ANOVA), followed by Scheffé’s post hoc test for multiple comparisons. All results are expressed as the mean ± standard deviation.

## 3. Results

### 3.1. Isolation and Molecular Identification of Strain DCP04

**Figure 1:**
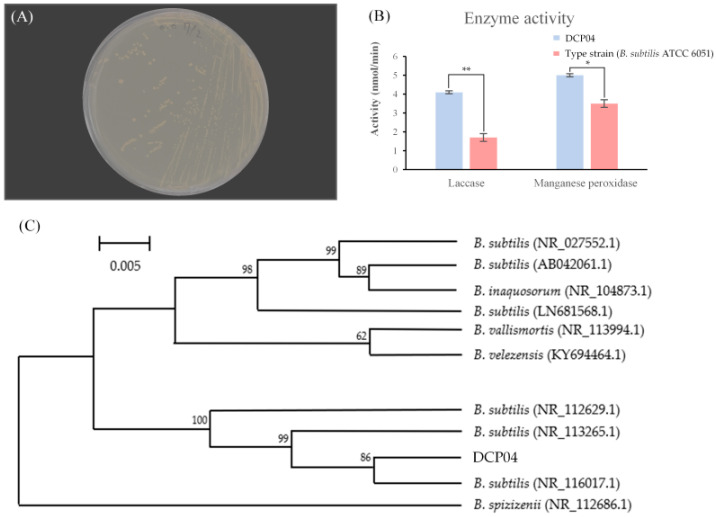
Isolation, identification, and enzymatic characterization of *B. subtilis* DCP04. (**A**) A clear halo zone formed by strain DCP04 on BSAM containing 0.1% LDPE powder. (**B**) A neighbor-joining phylogenetic tree based on 16S rRNA gene sequences, showing the relationship between strain DCP04 and reference *B. subtilis* strains. (**C**) Laccase and MnP activities of strain DCP04 compared to the type strain (*B. subtilis* ATCC 6051). Data are presented as mean ± SD from three independent experiments (*n* = 3). An asterisk (*) denotes a significant difference (*p* < 0.05), and double asterisks (**) denote a highly significant difference (*p* < 0.01) when compared to the type strain.

To isolate bacteria capable of degrading LDPE, cheonggukjang samples were screened on a basal salt agar medium (BSAM) containing LDPE powder as the sole carbon source. The isolate designated DCP04 formed distinct, transparent halo zones around its colonies, indicating its potential to utilize LDPE (Figure 1A).

For precise taxonomic classification, the 16S rRNA gene of strain DCP04 was sequenced. A BLASTn analysis revealed that the sequence shared 99.74% nucleotide identity with *Bacillus subtilis* strain BCRC 10255 (accession no. NR_116017.1). Furthermore, a neighbor-joining phylogenetic analysis confirmed that strain DCP04 robustly clusters within the *B. subtilis* clade, strongly supporting its identification as a strain of *Bacillus subtilis* (Figure 1B).

### 3.2. Probiotic and Safety Profile of Strain DCP04

The safety and probiotic-related characteristics of *B. subtilis* DCP04 were evaluated (Table 1). The strain exhibited no clearing zone on sheep blood agar, a result defined as *γ*-hemolysis (gamma-hemolysis), confirming its non-hemolytic nature. In contrast, the *β*-hemolytic positive controls produced clear lytic zones.

The strain displayed a moderate auto-aggregation ability (36.16 ± 0.4%). Its co-aggregation ability was pathogen-dependent; it co-aggregated moderately with *S. aureus* (35.58 ± 0.03%) and *P. aeruginosa* (25.52 ± 0.17%), but showed minimal co-aggregation with *E. coli* (5.05 ± 0.25%). These results, which are well above the typical <10% baseline for low aggregation, suggest a significant adhesive capacity [38,39]. Furthermore, strain DCP04 tested negative for both lysine and arginine decarboxylase activities, indicating it does not produce the corresponding biogenic amines under the tested conditions.

### 3.3. Enzyme Activities

To investigate the enzymatic basis for LDPE degradation, the activities of two key extracellular enzymes, laccase and MnP, were quantified and compared to the type strain, *B. subtilis* ATCC 6051. The DCP04 strain exhibited significantly higher enzymatic activities (Figure 1C). Its laccase activity was approximately 4.1 nmol/min, more than double that of the type strain. Similarly, its MnP activity was approximately 5.0 nmol/min, markedly exceeding that of the type strain. These elevated enzyme levels strongly suggest that *B. subtilis* DCP04 has the potential for effective LDPE biodegradation.

### 3.4. Adsorption of LDPE by B. subtilis DCP04

**Figure 2:**
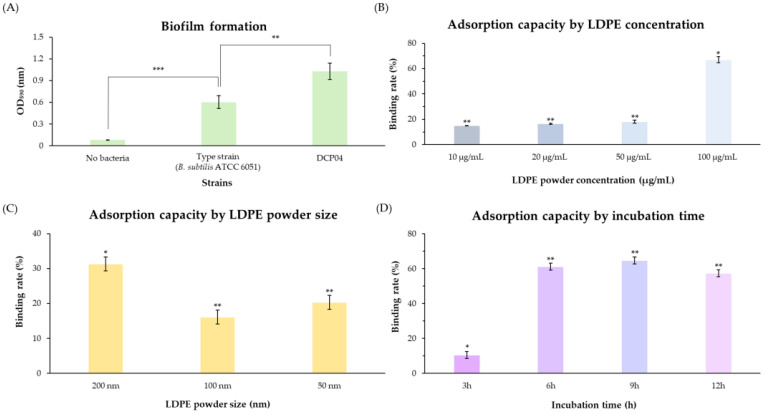
Biofilm formation and quantitative analysis of LDPE nanoparticle adsorption. (**A**) Quantification of biofilm formation by *B. subtilis* DCP04 strain. (**B**–**D**) NTA-based quantification of LDPE nanoparticle adsorption efficiency, showing the effects of (**B**) particle concentration, (**C**) particle size, and (**D**) incubation time. All quantitative data are presented as mean ± SD from three independent experiments (n = 3). An asterisk (*) denotes a significant difference (*p* < 0.05), double asterisks (**) denote a highly significant difference (*p* < 0.01), and triple asterisks (***) denote a significant difference (*p* < 0.001) when compared to the type strain.

The ability of *B. subtilis* DCP04 to adhere to surfaces, a prerequisite for particle interaction, was first evaluated. The strain demonstrated a strong capacity for biofilm formation in 96-well plates, reaching an absorbance value (OD_590_) of approximately 1.0–1.1 after 24 h (Figure 2A). This level of biofilm production is substantially higher than that reported for the type strain, *B. subtilis* ATCC 6051 (typically OD_590_ 0.4–0.8), suggesting a superior adhesive capability [40,41].

The efficiency of LDPE NP adsorption by *B. subtilis* DCP04 was quantified using NTA. The results demonstrated that the adsorption process was dependent on NP concentration, particle size, and incubation time. The adsorption rate exhibited a clear dose-dependent relationship, increasing significantly from approximately 10% at an NP concentration of 10 µg/mL to nearly 65% at 100 µg/mL (Figure 2B). Adsorption was also influenced by particle size, with the largest particles (200 nm) showing the highest binding rate (~32%), compared to smaller particles of 100 nm (~20%) and 50 nm (~15%) (Figure 2C). The interaction was also time-dependent; the binding rate increased from ~15% at 3 h to over 60% after 6 h, at which point the adsorption appeared to reach a plateau (Figure 2D).

**Figure 3:**
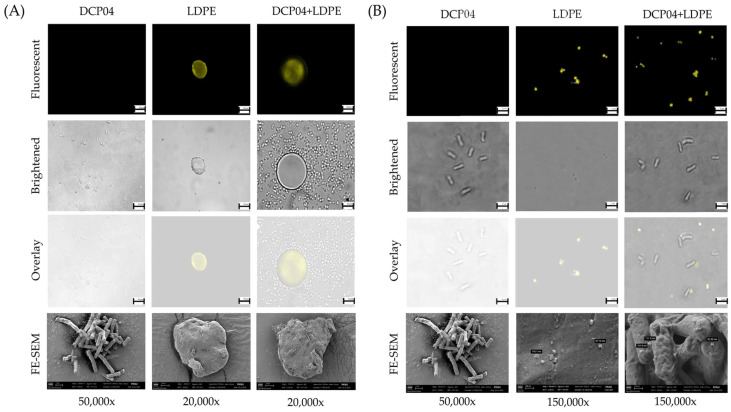
Representative micrographs visualizing the interaction between *B. subtilis* DCP04 and LDPE particles. (**A**) CLSM and FE-SEM images showing extensive colonization of bacterial cells on a micro-sized (25 μm) LDPE particle. (**B**) CLSM and FE-SEM images showing the adhesion of nano-sized (200 nm) LDPE particles to bacterial cells. Scale bars are included in each micrograph.

Direct visualization confirmed the physical interaction between the bacteria and LDPE particles. FE-SEM revealed extensive colonization of *B. subtilis* DCP04 on the surface of micro-sized LDPE particles, with cells forming dense clusters (Figure 3A). The strain also adhered effectively to nano-sized LDPE particles, underscoring its versatile adhesion capability (Figure 3B). CLSM further corroborated these findings, showing the co-localization of fluorescently stained LDPE particles with bacterial cells.

### 3.5. Biodegradation of LDPE Films by B. subtilis DCP04

#### 3.5.1. Weight Loss and Surface Morphology Analysis

**Figure 4:**
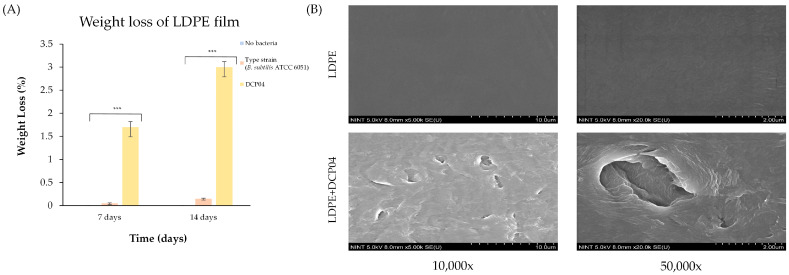
Biodegradation of LDPE films by *B. subtilis* DCP04. (**A**) Percentage weight loss of films after 14 days of incubation. (**B**) Representative SEM micrographs of LDPE film surfaces at 10,000× (**left**) and 50,000× (**right**) magnification. The upper panels show the smooth surface of an untreated control film, while the lower panels show the eroded and pitted surface of a film treated with strain DCP04. Data are presented as mean ± SD from three independent experiments (*n* = 3). An asterisk (***) indicates a significant difference (*p* < 0.05) compared to the ‘No bacteria’ control group.

The biodegradative capability of the DCP04 strain was first quantified by measuring the weight loss of LDPE films after a 14-day incubation period. A significant difference in weight loss was observed among the experimental groups (Figure 4A). Films incubated with *B. subtilis* DCP04 exhibited a substantial weight reduction of 2.92 ± 0.27%. In contrast, films incubated with the type strain (*B. subtilis* ATCC 6051) or in the bacteria-free control medium showed negligible weight loss (0.20 ± 0.03% and 0%, respectively). These findings indicate that, under the tested conditions, *B. subtilis* DCP04 possesses a unique capability to degrade LDPE films.

Changes in the surface morphology of the LDPE films were visualized using SEM (Figure 4B). The control films (no bacteria) displayed a smooth, dense, and featureless surface at both low and high magnifications, reflecting the pristine state of the polymer. In stark contrast, films incubated with the DCP04 strain showed profound surface degradation. The surface was characterized by the formation of numerous cavities, pits, and a general roughening. Higher magnification revealed large, irregularly shaped pores and erosion zones, providing clear visual evidence of substantial microstructural damage induced by bacterial activity.

#### 3.5.2. Analysis Chemical Modifications by FT-IR

**Figure 5:**
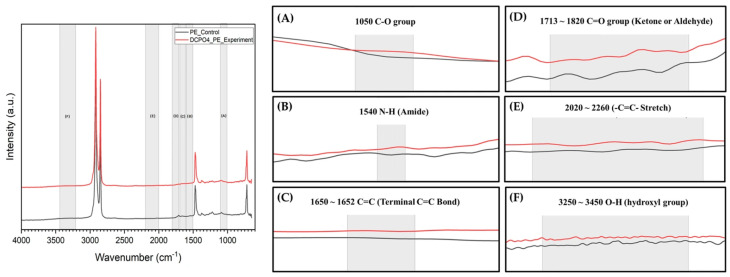
FT-IR analysis of chemical modifications on LDPE films. The spectra compare an untreated control film with a film after 14-day incubation with *B. subtilis* DCP04. Key spectral regions showing chemical alterations are highlighted: (**A**) C–O stretching, (**B**) Amide, (**C**) alkenes, (**D**) C=O stretching (carbonyl), (**E**) C≡C stretching region, and (**F**) O–H stretching (hydroxyl). The appearance of these functional groups indicates significant oxidative alteration of the polymer surface.

To investigate the chemical changes on the LDPE film surface following biodegradation, FT-IR spectroscopy was performed. The spectra of DCP04-treated films revealed the appearance of several new or intensified absorption bands compared to the untreated control, indicating significant chemical modification (Figure 5).

The most pronounced changes were indicative of extensive oxidation. A broad and strong absorption band emerged in the high wavenumber region (3250–3450 cm^−1^), which is characteristic of O–H stretching vibrations. This provides clear evidence for surface hydroxylation and the accumulation of hydroxylated byproducts from microbial activity (Figure 5F). Complementing this, a new peak cluster appeared between 1713 and 1820 cm^−1^, attributable to C=O stretching from various carbonyl functional groups (e.g., ketones, aldehydes, carboxylic acids). The formation of these carbonyls is unequivocal evidence of oxidative chain scission of the polyethylene backbone (Figure 5D). Further supporting the oxidation narrative, a distinct peak intensified around 1050 cm^−1^, corresponding to C–O stretching in alcohol or ether linkages (Figure 5A).

In addition to oxidative changes, other modifications were observed. The region around 1650 cm^−1^ showed increased absorbance, which can be attributed to the formation of C=C double bonds (alkenes) resulting from polymer chain dehydrogenation or elimination reactions during degradation (Figure 5C). Concurrently, the appearance of a shoulder peak at ~1540 cm^−1^ suggest the presence of residual bacterial biofilm or enzymes on the film surface (Figure 5B). Subtle changes were also detected in the 2020–2260 cm^−1^ region, which may indicate the formation of a small number of alkyne (C≡C) via complex degradation pathways (Figure 5E).

## 4. Discussion

This study reveals the dual functionality of *Bacillus subtilis* DCP04, a novel strain isolated from Cheonggukjang, demonstrating both a remarkable capacity to adsorb and biodegrade low-density polyethylene (LDPE). While the biodegradation of plastics by *Bacillus* species has been documented, this work provides, to our knowledge, the first comprehensive report on the significant adsorptive capabilities of a *B. subtilis* strain against LDPE nanoparticles, expanding the scope of probiotic-based mitigation strategies beyond conventional lactic acid bacteria [21,42].

The strong adsorptive performance of DCP04 is likely attributed to its superior biofilm-forming capabilities. Our results showed that DCP04 produced a substantially denser biofilm than the type strain, a key factor in mediating microbial adhesion to hydrophobic surfaces like plastic [43,44]. The formation of biofilms, rich in extracellular polymeric substances (EPS), can alter the plastic’s surface properties, increasing hydrophilicity and creating a conditioned interface for stable cell attachment and particle sequestration [38,45]. Intriguingly, our data did not show a clear trend of increased adsorption for smaller nanoparticles, a finding that differs from some previous reports [35]. This could suggest a complex interplay between particle surface chemistry, aggregation behavior in the medium, and the specific binding mechanisms of DCP04’s EPS, warranting further investigation. A key finding of this study is the remarkable capacity of DCP04 strain to adsorb LDPE nanoparticles (up to 65%), a functionality directly linked to its superior biofilm-forming capabilities. This mechanism is strongly supported by recent literature highlighting *B. subtilis* as a potent biosorbent for plastics. For instance, several studies have demonstrated the high efficiency of *B. subtilis* biofilms in removing polystyrene plastics, with reported removal rates ranging from 80% to over 90%, primarily through surface adsorption mediated by EPS [42,43]. The performance of DCP04 strain, which reached an adsorption efficiency of over 60% in just 6 h, is therefore consistent with the high efficiencies reported for this species and appears notably faster than other reported strains, such as the *Bacillus* sp. described by previous studies, which required 24 h to achieve a 55.4% removal rate. Furthermore, this high efficiency is particularly noteworthy when compared to the 20-50% adsorption rates typically reported for lactic acid bacteria [35]. Collectively, these findings underscore that DCP04 strain is not merely capable of plastic adsorption but is a highly efficient and rapid sequestrant. This positions *Bacillus* species, particularly food-derived strains like DCP04, as potent and compelling candidates for developing next-generation probiotic strategies to mitigate microplastic exposure, expanding the scope beyond conventional probiotics.

Beyond adsorption, the significant biodegradation of LDPE films by DCP04 was substantiated by multiple lines of evidence. The observed weight loss (2.92%) and the severe surface erosion seen in SEM images were mechanistically explained by the strain’s enzymatic machinery. Previous studies have documented PE degradation by *Bacillus* strains from various environments. One report showed a weight loss of up to 6.4% after a 40-day incubation period [46], while a more recent study observed an 8.9% weight loss of LDPE by *Bacillus cereus*, though this was achieved over a 60-day period [47]. While the total degradation percentages in these longer-term studies are higher, DCP04 demonstrates a markedly higher degradation rate, accomplishing a substantial reduction in a short period. This potency is likely attributed to the significantly higher laccase and manganese peroxidase activities observed in DCP04, suggesting an immediate enzymatic attack on the polymer backbone. DCP04 produced significantly higher levels of laccase and MnP, enzymes known to initiate polymer degradation through oxidative attack [38]. This enzymatic oxidation was unequivocally confirmed by FT-IR analysis. The appearance of new carbonyl (C=O, ~1730 cm^−1^) and hydroxyl (O-H, ~3400 cm^−1^) groups, coupled with the decreased intensity of C–H bonds (2850–2920 cm^−1^), provides a clear chemical fingerprint of polymer chain scission and oxidation, consistent with established biodegradation pathways [48,49].

The dual capacity for surface adsorption and enzymatic degradation positions DCP04 strain as a highly promising probiotic candidate for mitigating the in vivo risks of MP and NP exposure. While previous studies have highlighted the ability of *Lactobacillus* strains to bind and enhance the excretion of microplastics, our findings suggest that DCP04 strain could offer an additional benefit: the potential for partial degradation within the GI- tract [16]. This is particularly relevant given the known antioxidant, anti-inflammatory, and gut barrier-enhancing properties of other *B. subtilis* strains, which could further help counteract the toxicological effects of ingested plastics [50,51].

While this study provides compelling in vitro evidence, several limitations must be acknowledged before considering clinical applications. All experiments were conducted under controlled laboratory conditions, which may not fully represent the complex and dynamic environment of the human gut. While this study provides compelling in vitro evidence, its main limitations must be clearly acknowledged to frame the context for future work. The primary limitation is that all experiments were conducted under controlled laboratory conditions, which do not fully represent the complex and dynamic ecosystem of the human GI tract. Factors such as pH fluctuations, digestive enzymes, and bile salts could significantly influence the strain’s activity and survival. Therefore, while the initial safety profile of DCP04 is promising (non-hemolytic, no biogenic amine production), further characterization is essential. Future work must thoroughly evaluate its tolerance to gastric acid and bile salts to ensure its survival through the GI tract, and a comprehensive antibiotic susceptibility profile is required to rule out transferable resistance genes. Furthermore, this study focused exclusively on LDPE; the efficacy of DCP04 against other prevalent polymers like polyethylene terephthalate (PET) and polystyrene (PS) remains unknown. Finally, a metabolomic analysis to identify the specific byproducts of LDPE degradation is imperative to ensure they are non-toxic to the host. These comprehensive assessments are critical next steps that must be prioritized before any clinical application can be considered [52]. Therefore, future research should prioritize validating these findings in simulated GI models and subsequently in in vivo animal studies to assess the strain’s survival, safety, and efficacy in a biological system.

Looking ahead, a deeper mechanistic understanding is required. Metabolomic analyses using techniques like HPLC and GC-MS are imperative to identify the specific intermediate products of LDPE degradation. This would not only elucidate the complete metabolic pathway but also, crucially, allow for the verification of the biosafety of these byproducts, ensuring they are non-toxic to the host [53]. Furthermore, the efficacy of DCP04 should be tested against other common polymer types (e.g., PET, PS) to determine the breadth of its activity. Investigating potential synergistic effects by co-culturing DCP04 with other beneficial probiotic strains could also uncover enhanced plastic-eliminating functionalities, paving the way for next-generation probiotic formulations to combat the growing health threat of MPs and NPs.

## 5. Conclusions

In conclusion, this study demonstrates that *Bacillus subtilis* DCP04, a novel strain isolated from the Korean fermented food cheonggukjang, possesses a significant dual capacity to both adsorb and biodegrade LDPE. The strain exhibited high adsorptive efficiency for LDPE nanoparticles (up to 65%), a performance comparable or superior to previously studied lactic acid bacteria. Furthermore, it demonstrated a rapid biodegradative capability, causing a 2.92% weight loss in LDPE films in just 14 days, a rate significantly faster than that of other reported *Bacillus* strains. These findings present *B. subtilis* DCP04 as a promising probiotic candidate for a novel in vivo strategy to mitigate the accumulation of micro- and nanoplastics. The use of a safe strain derived from a traditional fermented food source makes this a particularly compelling approach. However, comprehensive in vivo studies are essential to validate its safety, efficacy, and survival within the GI tract before any clinical application can be considered. Future research should also expand to assess its efficacy against other common polymers, such as PET and PS, identify the byproducts of degradation to ensure their non-toxicity, and explore potential synergistic combinations with other probiotic strains. Ultimately, this work paves the way for developing novel, food-derived biological agents to address the urgent health challenges posed by plastic pollution.

## Figures and Tables

**Table 1:** Safety and probiotic-related characteristics of *B. subtilis* DCP04.

Strain	Auto- Aggregation (%)	Co-Aggregation (%)			Biogenic Amine		Hemolysis
		*E. coli*	*S. aureus*	*P. aeruginosa*	Lysine Decarboxylase	Arginine Decarboxylase	
*B. subtilis* DCP04	36.16 ± 0.40	5.05 ± 0.25	25.58 ± 0.03	25.52 ± 0.17	-	-	-

In the tables, “-” indicates a “negative”. Data are presented as Mean ± SD.

## Data Availability

The original contributions presented in this study are included in the article. Further inquiries can be directed to the corresponding author.

## Abbreviations

The following abbreviations are used in this manuscript:

BSAM	Basal salt agar medium
CLSM	Confocal laser scanning microscopy
FE-SEM	Field emission scanning electron microscope
FT-IR	Fourier-transform infrared spectroscopy
GI	Gastrointesinal
LDPE	Low-density polyethylene
MnP	Manganese peroxidase
MP	Microplastic
NP	Nanoplastic
NTA	Nanoparticle tracking analysis
PBS	Phosphate-buffered saline
TSB	Tryptic soy broth
